# Fiber type-specific expression of LACTB leverages a function in oxidative metabolism

**DOI:** 10.1007/s00418-026-02476-8

**Published:** 2026-04-18

**Authors:** Kirsi-Marja Alanen, Rabah Soliymani, Jaakko Sarparanta, Satu Kuure, Kirsi Sainio, Zydrune Polianskyte, Muhammad Yasir Asghar, Ehsan Zangene, Annunziata Cascone, Maciej Lalowski, Peter Hackman, Johan Lundin, Dan Lindholm, Ove Eriksson

**Affiliations:** 1 Biochemistry and Developmental Biology, Medicum, Faculty of Medicine, University of Helsinki, Biomedicum Helsinki 1, Haartmaninkatu 8, 00290 Helsinki, Finland; 2 Meilahti Proteomics Unit, Faculty of Medicine, HiLIFE, University of Helsinki, Helsinki, Finland; 3 Folkhälsan Research Center, Helsinki, Finland; 4 GM-Unit, HiLIFE, University of Helsinki, Helsinki, Finland; 5 Stem Cells and Metabolism Research Program Unit, HiLIFE, University of Helsinki, Helsinki, Finland; 6 Minerva Foundation Institute for Medical Research, Biomedicum Helsinki 2, Helsinki, Finland; 7 Institute for Molecular Medicine Finland (FIMM), HiLIFE, University of Helsinki, Helsinki, Finland; 8 Department of Global Public Health, Karolinska Institutet, Stockholm, Sweden; 9 Department of Biomedical and Clinical Sciences, Linköping University, Linköping, Sweden; 10 Department of Gene Expression, Institute of Molecular Biology and Biotechnology, Faculty of Biology, Adam Mickiewicz University, 61-614 Poznan, Poland

**Keywords:** Mitochondria, LACTB, Serine protease, Type I muscle fiber, Type II muscle fiber, Muscle differentiation

## Abstract

Skeletal muscle is composed of type I and type II fibers, each characterized by specific metabolic machinery. LACTB is a conserved mitochondrial protein implicated in lipid utilization and tumorigenesis, but its precise function in the cellular metabolism remains unclear. To gain novel insight into the functional role of LACTB, we investigated skeletal muscle to determine whether LACTB is segregated by fiber type. The expression of LACTB was determined by immunohistochemistry (IHC) and immunoblotting in skeletal muscle from healthy human subjects and the laboratory rat. The specificity of the antibody was assessed using recombinant human LACTB protein and endogenous LACTB in isolated mitochondria. IHC results were validated in a cellular model of myoblast differentiation using the C2C12 and L6 cell lines. The results demonstrated that LACTB is highly enriched in adult type I muscle fibers. During development, LACTB expression commences in type I primary myotubes at the time of their formation around week 20 of gestational age. LACTB expression in myoblasts is low but increases rapidly upon the induction of myotube differentiation. We conclude that LACTB plays a distinct role in mitochondria of type I fibers, most likely acting in oxidative metabolism related to energy use from lipids. This defines LACTB as a mitochondrial marker for type I fibers.

**Supplementary Information:**

The online version contains supplementary material available at 10.1007/s00418-026-02476-8.

## Background

LACTB is a mammalian mitochondrial protein implicated in lipid turnover and tumorigenesis (Cascone et al. 2022; Li et al. 2022). LACTB is located in the mitochondrial intermembrane space and can polymerize into membrane-attached helical filaments (Polianskyte et al. 2009), the structure of which was recently resolved by cryo-electron microscopy (Bennett et al. 2022; Zhang et al. 2022). LACTB shares sequence similarity with the bacterial penicillin-binding proteins (PBPs) involved in peptidoglycan synthesis, and the amino acids required for catalytic activity are conserved in LACTB (Peitsaro et al. 2008). However, the biochemical function and physiological substrate(s) of LACTB remain unclear.

Several system-level studies aimed at uncovering genetic pathways dysregulated in obesity-related traits have revealed that LACTB influences sterol, fatty acid, and lipoprotein metabolism (Schadt et al. 2005; Chen et al. 2008; Chatterjee et al. 2009; Yang et al. 2009; Teslovich et al. 2010; Willer et al. 2013), as shown in a LACTB transgenic mouse model having mild obesity (Chen et al. 2008; Yang et al. 2009). A study by Keckesova et al. (2017) revealed that LACTB exerts tumor suppressive and antiproliferative effects in breast cancer cells. Subsequently, LACTB was shown to be downregulated in several human tumors (Li et al. 2022). although upregulation of LACTB has been reported for pancreatic cancer (Xie et al. 2021) and nasopharyngeal carcinoma (Peng et al. 2021). To clarify the role of LACTB in tumorigenesis, a deeper understanding of LACTB’s physiological function is needed.

Currently, only sparse information on the function of LACTB in skeletal muscle is available. In an early study of human muscle, *LACTB* messenger RNA (mRNA) expression was found to increase during a hyperinsulinemic clamp (Rome et al. 2003). Recently, a study in mice revealed a shift in the polymerization state of LACTB following exercise adaptation (Gonzalez-Franquesa et al. 2021), and a study of epigenetic responses to exercise in mice reported upregulation of *LACTB* mRNA (Chambers et al. 2025). However, these studies did not address potential differences between fiber types. The differences in skeletal muscle fiber types have been extensively characterized from both a molecular and functional perspective. To test whether the metabolic differences between the fiber types were tied to a difference in LACTB expression level, we studied human adult and fetal skeletal muscle. Our results revealed that LACTB plays a distinct role in the maturation of muscle mitochondria, most likely related to a function in oxidative metabolism specific for type I muscle fibers.

## Materials and methods

### Reagents

Reagents were purchased from Sigma-Aldrich (St. Louis, MO, USA), unless otherwise stated.

### Antibodies

To generate polyclonal anti-LACTB antibodies, we selected an 18-amino-acid target epitope close to the N-terminus of fully processed human LACTB (hLACTB) and rat LACTB (rLACTB) (Polianskyte et al. 2009) (Fig. S1, panel a). This epitope is part of a flexible segment located outside the beta-lactamase backbone (Bennett et al. 2022; Zhang et al. 2022). Previous analyses by mass spectrometry have indicated that this segment does not carry any post-translational modifications (Polianskyte et al. 2009). Amino acid sequence searches using the Basic Local Alignment Search Tool (BLAST, National Center for Biotechnology Information, Bethesda, MD, USA) revealed that the sequence was unique to LACTB. Immunization and antibody purification were performed by New England Peptide (Gardner, MA, USA). In brief, for each target epitope (hLACTB and rLACTB), two rabbits (New Zealand White) were immunized three times with synthetic peptides corresponding to the epitope sequences. Pre-immune serum was collected for each rabbit. Two bleedings of 50 mL antiserum were used for affinity purification against the immobilized target epitope.

Commercially purchased primary antibodies were as follows: anti-cytochrome oxidase subunit IV (COX-IV), Proteintech (66110-1-Ig; Proteintech Group, Inc, Rosemont, IL, USA); anti-carnitine palmitoyl transferase 1B (CPT1B), Proteintech (22170-1-AP); anti-glyceraldehydephosphate dehydrogenase (GAPDH), Cell Signaling Technology (Leiden, Netherlands); anti-isocitrate dehydrogenase 2 (IDH-2), Abcam (ab55271; Abcam, Waltham, MA, USA); anti-isocitrate dehydrogenase 3 (IDH-3), Proteintech (68199-1-Ig); anti-Ki-67, Abcam (ab279653); anti-laminin beta 1, Merck (Darmstadt, Germany) (MAB1921b), anti-myosin heavy chain 1 (MYH1), Proteintech (67299-1-Ig); anti-myosin heavy chain 2 (MYH2), Proteintech (66212-1-Ig); anti-myosin heavy chain 3 (MYH3), Invitrogen (PA5-72848; Invitrogen, Carlsbad, CA, USA); anti-myosin heavy chain 7 (MYH7), Invitrogen (TH81); anti-myosin heavy chain 8 (MYH8), Invitrogen (PA5-72,846); anti-myoblast determination protein 1 (MyoD1), Abcam (Ab16148); anti-paired box protein 7 (Pax-7), Abcam (ab218472); anti-phosphatidyl serine decarboxylase (PISD), Abcam (ab236405); anti-slow twitch skeletal muscle troponin T (TnnT1), Proteintech (68631-1-Ig); and anti-voltage-dependent anion channel 1 (VDAC1), Merck (MABN504). Commercially purchased secondary antibodies were as follows: anti-mouse immunoglobulin G (IgG) horseradish peroxidase (HRP), Invitrogen (G-21040); donkey anti-rabbit IgG HRP, Thermo Fisher Scientific (A16035); goat anti-rabbit IgG Alexa-647, Thermo Fisher Scientific (A21245); and goat anti-mouse IgG Alexa-750, Thermo Fisher Scientific (A21037). Validation of the commercially purchased antibodies was provided in the technical specifications insert for each antibody. Literature references to the antibodies and Research Resource Identifiers are listed in Supplementary Table S1.

### Validation of anti-LACTB antibodies

To test the anti-hLACTB antibody, we used recombinant hLACTB protein added in incremental quantities to a protein extract of ML-1 human follicular thyroid carcinoma cells (Srinivasan et al. 2023). The results indicated that the anti-hLACTB antibody detected recombinant hLACTB protein added at quantities of 0.5, 1, and 2 ng, while the endogenous expression level of LACTB in ML-1 cells was below the detection limit (Fig. S1, panel b). Polymeric forms of LACTB could be seen as minor bands at higher molecular weight.

To test the anti-rLACTB antibody, mitochondria were prepared from rat heart, kidney, liver, brain, and skeletal muscle, after which proteins were separated by sodium dodecyl sulfate polyacrylamide gel electrophoresis (SDS-PAGE) for immunoblotting. The result showed that the anti-rLACTB antibody detected a band at about 54 kDa corresponding to the molecular mass of processed LACTB (Fig. S1, panel c). The apparent content of LACTB in heart and skeletal muscle was lower than that for the other tissues, probably because a substantial quantity of muscle mitochondria remains attached to the myofibrillar scaffold (Kelley et al. 2002). Bands were excised from SDS-PAGE gels at the position corresponding to 54 kDa and subjected to in-gel hydrolysis by trypsin, followed by analysis by mass spectrometry, which confirmed the presence of LACTB (Fig. S1, panel d).

Using the anti-rLACTB antibody on the recombinant hLACTB protein demonstrated no cross-reactivity against hLACTB. Likewise, using the anti-hLACTB antibody on mitochondrial proteins separated by SDS-PAGE revealed little or no cross-reactivity of the anti-hLACTB antibody for rLACTB protein (not shown).

To validate the expression data, we used a Multiple Tissue Expression Array to determine the relative levels of *LACTB* mRNA expression in 75 different human tissues and cancer cell lines. Hybridization with the 421-base-pair (bp) probe encompassing the catalytic -SISK- motif revealed that *LACTB* mRNA was expressed in all adult tissues (Fig. S1, panels e and f, Supplemental Table S2). The highest *LACTB* mRNA expression levels were found in the heart, skeletal muscle, liver, and kidney, while the lowest levels were found in the uterus, thyroid gland, and ovary. Fetal tissues contained lower levels of *LACTB* mRNA than the corresponding adult tissues. In agreement with findings from other studies (Keckesova et al. 2017), cancer cell lines contained low levels of *LACTB* mRNA (Supplementary Table S2).

### Preparation of tissues for immunohistochemical staining

Paraffin-embedded sections of human skeletal muscle were purchased from TissueArray.Com LLC, USA (Derwood, MD, USA). The catalogue numbers were as follows: HuFPT075 (normal human skeletal muscle), MC245c (normal human skeletal muscle), and BE01014a (normal human fetal tissue).

To collect rat tissue samples, male Wistar rats (200 g) under CO_2_ sedation were killed by decapitation, whereupon the musculus vastus lateralis was excised and fixed by immersion in a fixative containing 4% paraformaldehyde, 1% dimethyl sulfoxide (DMSO) in phosphate-buffered saline (PBS), pH 7.4. Fixation was allowed to proceed for 24 h at room temperature (RT). The samples were then transferred in a graded ethanol series to a final concentration of 70% ethanol and embedded in paraffin using a Logos Processing machine (Milestone, Valbrembo, Italy) with the standard program for tissues up to 5 mm thickness. Sections (4 µm) were cut using a Microm HM355S microtome (Thermo Fisher Scientific, Waltham, MA, USA).

### Immunohistochemical staining and digitization of images

Sections were incubated at 60 °C for 15–30 min to ensure proper adhesion. Sections were then deparaffinized and rehydrated in xylene and descending alcohol series. Heat-induced epitope retrieval was performed in 10 mM Tris-1 mM EDTA pH 9 for 20 min at 99 °C using the PT Module (epredia, Kalamazoo, MI, USA). Before applying the primary antibodies, the tissue sections were blocked with 10% goat serum in Tris-buffered saline containing 0.05% Tween 20 (TBST) for 15 min at RT.

Primary antibodies for single or double labeling experiments were diluted in 10% goat serum in TBST, whereupon tissue sections were incubated for 1 h at RT. Primary antibodies were visualized using secondary antibodies conjugated to the Alexa Fluor 647 or Alexa Fluor 750 fluorescent dye. Secondary antibodies were diluted 1:300 in TBST, and tissue sections were incubated for 30 min at RT. When indicated, 4′,6-diamidino-2-phenylindole (DAPI) was added together with the secondary antibody at a concentration of 1.6 µg/mL. ProLong Gold (Thermo Fisher Scientific) was used as an anti-fading mounting medium. 3,3’-Diaminobenzidine (DAB) was obtained from Thermo Fisher Scientific. Digital images of stained tissues were acquired using a whole-slide Pannoramic 250 FLASH III Digital Scanner (3DHISTECH Kft, Budapest, Hungary) equipped with a plan apochromat objective ×20 (NA 0.8) and a scientific sCMOS camera (PCO.edge 4.2, Excelitas Technologies Corp, Pittsburgh, PA, USA), resulting in a final pixel size of 0.325 μm. Fluorescent filters suitable for wavelengths of 365 nm (DAPI), 647 nm, and 750 nm were used. Figures were assembled using Microsoft PowerPoint (Microsoft, Redmond, WA, USA).

### Confocal microscopy

The images and z-stack series were captured using a Leica STELLARIS 8 FALCON (Leica Microsystems GmbH, Wetzlar, Germany) system equipped with HyD S detectors and an HC PL APO CS2 ×63/1.40 oil objective (Leica Microsystems) using the 647 and 750 nm laser lines. Parameter settings were kept the same during all the imaging processes. Images were captured and analyzed using Leica Application Suite X Software v.4.8.1.29271 (Leica Microsystems) and formatted using Fiji ImageJ software (v.2.1.0/1.53c; National Institutes of Health).

### Preparation of mitochondria

Mitochondria were prepared from rat tissues as described previously (Johans et al. 2005). Briefly, tissues of interest were resected and placed in isolation buffer containing 250 mM sucrose, 10 mM Hepes-K, and 1 mM EGTA, pH 7.4. Samples were cut into pieces and homogenized in a tight-fitting pestle for 1–2 min. Heart and skeletal muscle samples were homogenized with a T10 basix Ultra-Turrax disperser (IKA-Werke GmbH & Co., Staufen, Germany) for 30 s before homogenization. The resulting tissue homogenates were centrifuged at 750×*g* for 7.5 min, whereupon the supernatant was collected and re-centrifuged at 10,000×*g* for 10 min. The pellet was resuspended in isolation buffer and centrifuged for 10 min at 10,000×*g*. The resulting mitochondrial pellet was collected and resuspended in an approximately equal volume of isolation buffer. All preparative steps were performed at +4 °C. Protein concentration was measured by colorimetry using the Protein Assay Dye Reagent Concentrate (Cat# 3500-0006, Bio-Rade, Hercules, CA, USA). Mitochondria were solubilized in 4 M urea, and bovine serum albumin was used as the standard.

### Cell culture

C2C12 mouse myoblasts (ATCC CRL-1772, American Type Culture Collection, Manassas, VA, USA) were maintained in growth medium (pyruvate- and phenol red-free, high-glucose Dulbecco’s modified Eagle medium [DMEM] supplemented with GlutaMax, penicillin/streptomycin, and 20% fetal calf serum). For differentiation into myotubes, the cells were seeded on ultra-compliant gelatin hydrogels prepared as described by Jensen et al. (2020). Two days after plating, upon reaching confluency, differentiation medium (DMO; pyruvate- and phenol red-free, high-glucose DMEM supplemented with L-glutamine, penicillin/streptomycin, 2% heat-inactivated horse serum, and 10% Opti-MEM I) was added. Cells were differentiated for up to 14 days with daily medium changes. C2C12 cells were harvested for protein samples at the myoblast stage and at different time points during differentiation.

Confluent L6 rat myoblasts (ATCC CRL-1458) were differentiated in DMEM medium containing 2% FCS, supplemented with IGF-1, retinoic acid, and cytosine-1-β-D-arabinofuranoside (AraC) for 6 days. L6 cells were harvested after reaching confluence and at different time points during differentiation.

Human ML-1 follicular thyroid carcinoma cells (Schönberger et al. 2000) were grown in DMEM supplemented with 10% fetal bovine serum (FBS), 1% penicillin/streptomycin, and 1% L-glutamine. Cells were harvested for protein samples at 80% confluence.

The protein concentration of cultured cells was determined as described for isolated mitochondria.

### SDS-PAGE and immunoblotting

Isolated mitochondria or cultured cells were solubilized at a concentration of 4 µg protein/µL in Bolt sample buffer (Thermo Fisher Scientific) supplemented with 5% 2-mercaptoethanol. Samples containing 20 µg protein were heated for 10 min at 60 °C and loaded onto Bolt™ 5–14%, Bis–Tris Plus WedgeWell™ gels (Invitrogen, Carlsbad, CA, USA) and run for 60–90 min at 160 V. Proteins were transferred onto nitrocellulose membrane using a Bio-Rad Trans-Blot Turbo machine. Blots were incubated with primary antibodies for 12 h at 4 °C, followed by three washes for 10 min each with TBST, and incubation with secondary antibodies for 2 h at RT. Visualization of the antibodies was performed with the ECL Plus Western Blotting Substrate (Thermo Fisher Scientific). Immunoblots were scanned on a Bio-Rad ChemiDoc™ Touch Imaging System scanner.

### Mass spectrometry

Immunoreactive protein bands were excised from nitrocellulose membranes, reduced, and alkylated before trypsin digestion essentially as described in Vaarala et al. (2014). In brief, reduction and alkylation of proteins in excised bands was performed by incubation in a solution containing 5 mM tris (2-carboxyethyl) phosphine, 50 mM iodoacetamide, and 200 mM ammonium bicarbonate for 30 min in the dark. Sequencing Grade Modified Trypsin (Promega Corporation, Madison, WI, USA) dissolved in 50 mM ammonium bicarbonate was added to excised samples at a ratio of 1:50 (µg trypsin to µg sample protein) and incubated overnight at room temperature with continuous shaking. The resulting peptide mixture was diluted to 100 µL with 0.3% trifluoroacetic acid and concentrated using Pierce™ C18 reversed-phase tips (Thermo Fisher Scientific). Elution of peptides from the reversed-phase tips was performed with a solution containing 50% acetonitrile and 0.3% trifluoroacetic acid in double-distilled H_2_O. Eluted peptides were dried under vacuum and stored at −80 °C. Dried peptides were resuspended in 10 µL 0.3% TFA (solution A) and sonicated in a water bath for 1 min before injection into the mass spectrometer. Peptides were analyzed using nano-LC-Thermo Q Exactive HF (Thermo Fisher Scientific), as described in Srinivasan et al. (2025) with minor changes. After trapping, peptides were separated with a linear gradient of 60 min comprising 30 min from 3% to 30% of solution B (0.1% formic acid/80% acetonitrile), 5 min from 30% to 40% of solution B, and 4 min from 40% to 95% of solution B. Liquid chromatography–tandem mass spectrometry (LC–MS/MS) data acquisition was performed with the mass spectrometer resolution set to 140,000 and 15,000 for MS and MS/MS scans, respectively. Secondary ions were isolated with a window of 1.2 m/z. Maximum injection time values were set to 50 and 80 ms for MS and MS/MS, respectively.

Following LC–MS/MS data acquisition, raw files were analyzed by Proteome Discoverer version 2.5 (Thermo Fisher Scientific). Protein identification was performed against the reviewed UniProtKB/SwissProt protein database with taxonomy limited to rat and mouse sequences (releases 2023_01 with 8180/17139 entries) using the built-in SEQUEST HT and Mascot engines (Matrix Science Limited, London, UK). The following parameters were used in the searches: 5 ppm and 0.02 Da tolerance for MS and MS/MS, respectively; trypsin as digesting enzyme with one missed cleavage allowed; carbamidomethylation of cysteines as fixed modification; and oxidation of methionine oxidation and asparagine/glutamine deamidation as variable modifications; false discovery rate was set to less than 0.01; and a peptide minimum length of six amino acids.

### Oligonucleotides and dot blot analysis

Human LACTB complementary DNA (cDNA) (clone BC067288: pCMV-SPORT6.1-hLACTB) was purchased (I.M.A.G.E.). The human LACTB insert was amplified by polymerase chain reaction (PCR) with Phusion DNA-polymerase (Thermo Fisher Scientific), using the forward primer 5’-CACCATGTACCGGCTCCTGTCAAG-3’ and the reverse primer 5’-GTCAGCTCTGTCTTTATCAAATTC-3’. The purified PCR products were cloned into a linearized pENTR/SD/D-TOPO vector according to the manufacturer’s instructions (Invitrogen). All inserts were confirmed by sequencing. For dot blot analysis of tissue expression, a 421-bp DNA segment encompassing the -SXXK- signature motif (-SISK- in human LACTB protein) was amplified from the pENTR-hLACTB plasmid using the forward primer 5’-CACCATGAGAGCCATCGAGAGCAG-3’ and the reverse primer 5’-TTTCACCATCTTCAGGGCTTT-3’.

The 421-bp PCR product encompassing the -SXXK- signature motif was randomly labeled with [α-^32^P]dCTP using the Multiprime Labeling Kit (GE HealthCare Finland Oy, Helsinki, Finland). The resulting probe was hybridized with immobilized mRNA on a Multiple Tissue Expression Array (BD Clontech) containing samples from different human tissues and cancer cell lines. Poly A+ RNA samples on each Multiple Tissue Expression Array were normalized to the mRNA expression levels of eight different housekeeping genes. Hybridization was performed using the ExpressHyb kit according to the manufacturer’s instructions (BD Clontech, now Takara Bio, San Jose, CA, USA). The hybridized array was exposed to a phosphorimager plate, and radioactivity was quantified using the MacBAS v2.5 software package (Fujifilm Nordic AB, Espoo, Finland).

## Results

### Human adult skeletal muscle

**Fig. 1:**
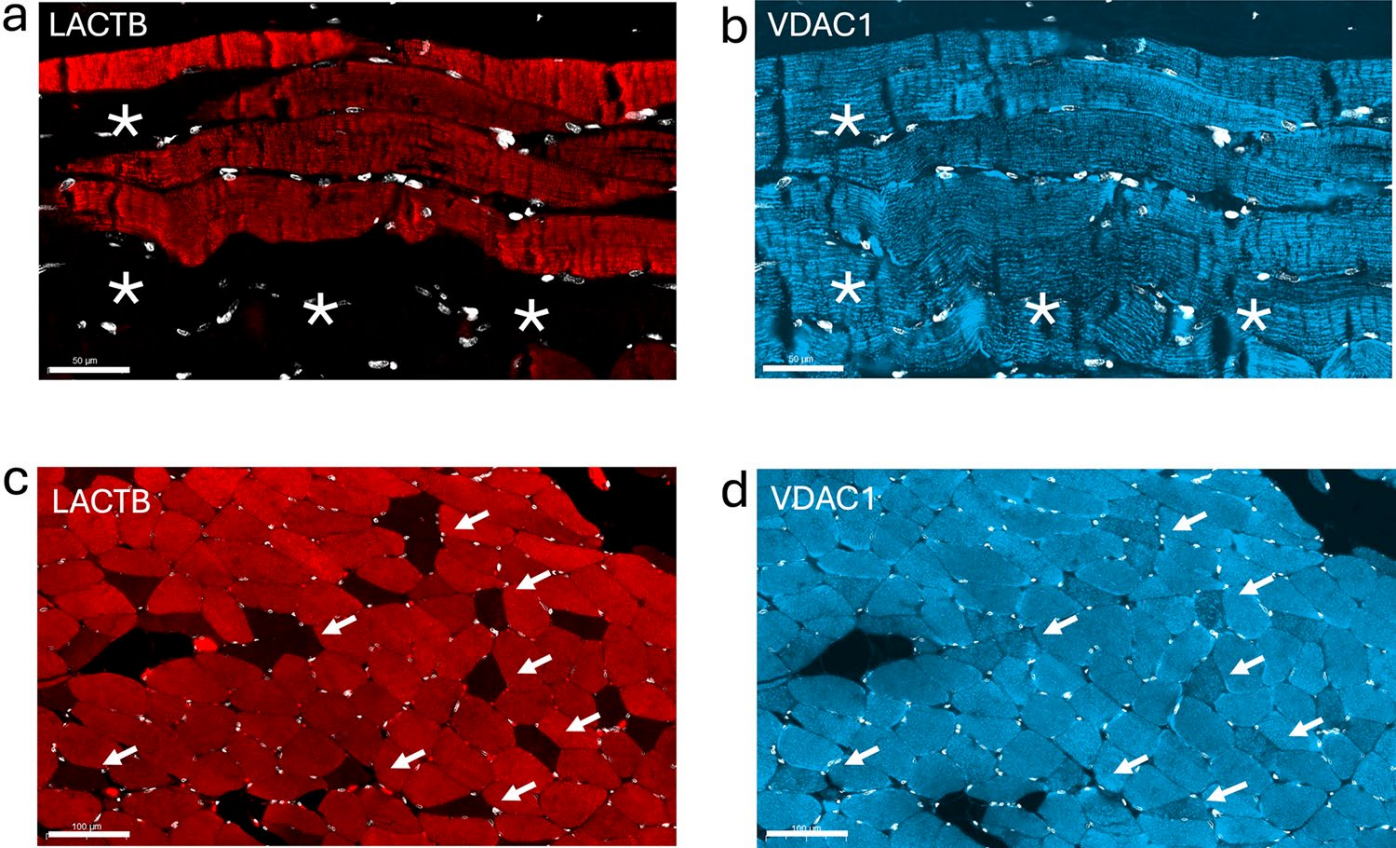
Visualization of LACTB and VDAC1 in adult skeletal human muscle. Paraffin sections were stained with antibodies against hLACTB (red) and VDAC1 (blue) as described in the Materials and Methods section. Nuclei stained with 4′,6-diamidino-2-phenylindole (DAPI) are shown in white. Panels **a** and **b** Identical areas of the musculus quadriceps femoris. Scale bars = 50 µm. Panels **c** and **d** Identical areas of the masseter muscle. Scale bars = 100 µm. Fibers negative for LACTB are indicated with *asterisks* in panel **a**, and with *arrowheads* in **c**. Musculus quadriceps and musculus masseter were from a 21- and a 34-year-old female, respectively

To investigate the expression of LACTB in adult human skeletal muscle, we performed immunohistochemical staining (IHC) of paraffin-embedded and sectioned samples of the quadriceps muscle using the anti-hLACTB antibody. Prior to use, the specificity of the antibody was validated by immunoblotting and mass spectrometry as detailed in the Materials and Methods section (Fig. S1). As shown in Fig. 1 panel a, the anti-hLACTB antibody stained numerous muscle fibers, but we noted some areas that showed no detectable LACTB staining despite the presence of nuclei in these areas. To visualize mitochondria, we stained the section with an antibody against the voltage-dependent anion channel 1 (VDAC1), and the results revealed that mitochondria were also present in the LACTB-negative areas (Fig. 1, panel b). Double IHC with anti-hLACTB and anti-laminin B1 antibodies to display the endomysium, and visualization of the anti-hLACTB antibody with 3,3-diaminobenzidine (DAB) for bright-field analysis yielded consistent results (Fig. S2, panels a and b). To corroborate this finding, we stained sections of the masseter muscle with the anti-hLACTB and anti-VDAC1 antibodies (Fig. 1, panels c and d), which revealed a mixed population of LACTB-positive and LACTB-negative fibers. These results demonstrate that LACTB is expressed in a subset of skeletal muscle fibers.

Analysis of the quadriceps sections with confocal microscopy showed that LACTB was distributed evenly over the whole volume of the LACTB-positive muscle fibers with a preference for I-band-associated mitochondria as defined by Glancy et al. (2015) (Fig. S2, panel c, Supplemental Video 1). The longitudinal spacing of the mitochondrial stacks was about 1.9 µm, in agreement with previous findings.

**Fig. 2:**
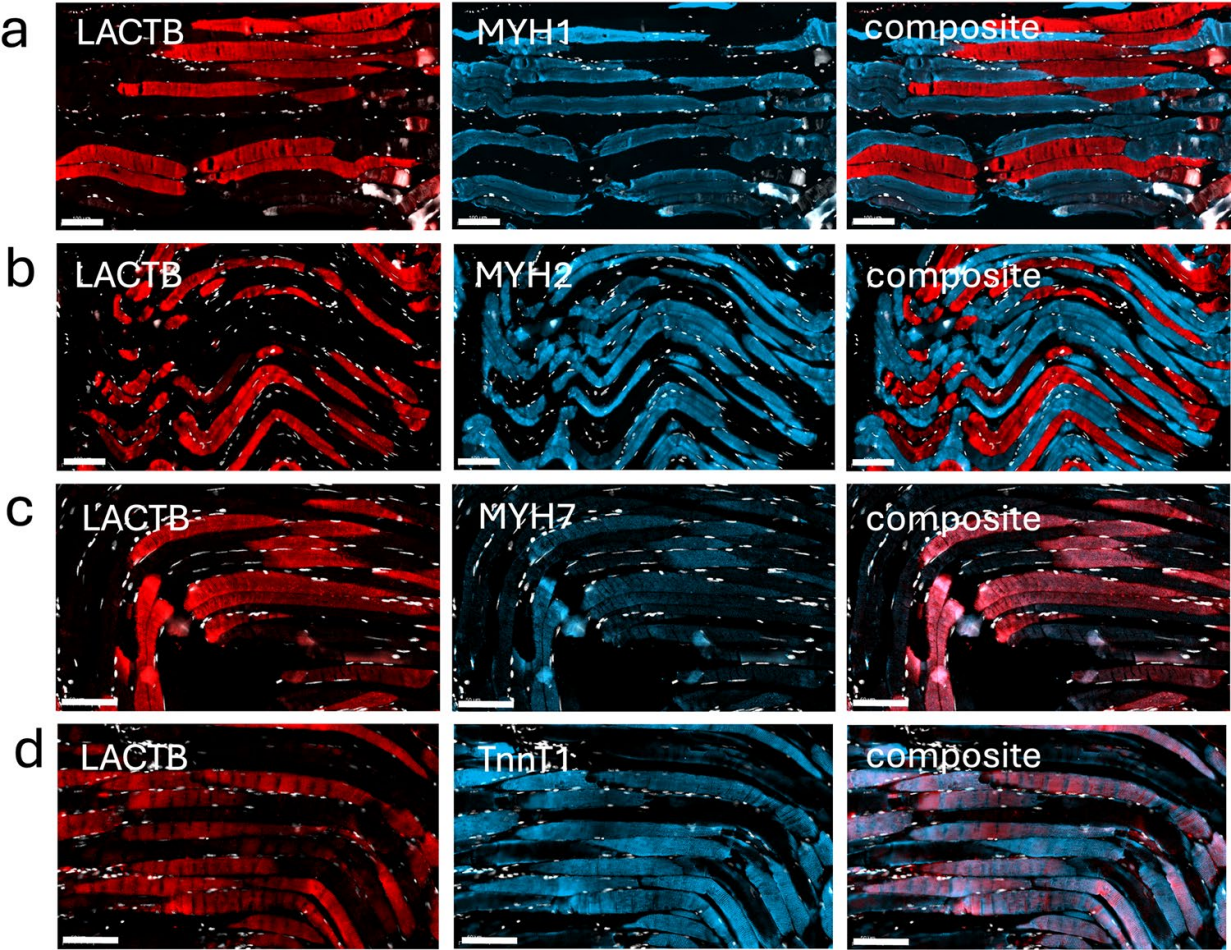
Visualization of LACTB and structural proteins in adult human muscle. Rows **a** and **b** LACTB (red) and markers for fast type II fibers, MYH1 and MYH2 (blue). Rows **c** and **d** LACTB (red) and markers for slow type I fibers, MYH7 and TnnT1 (blue). Nuclei stained with DAPI are shown in white. Scale bars = 100 µm

**Fig. 3:**
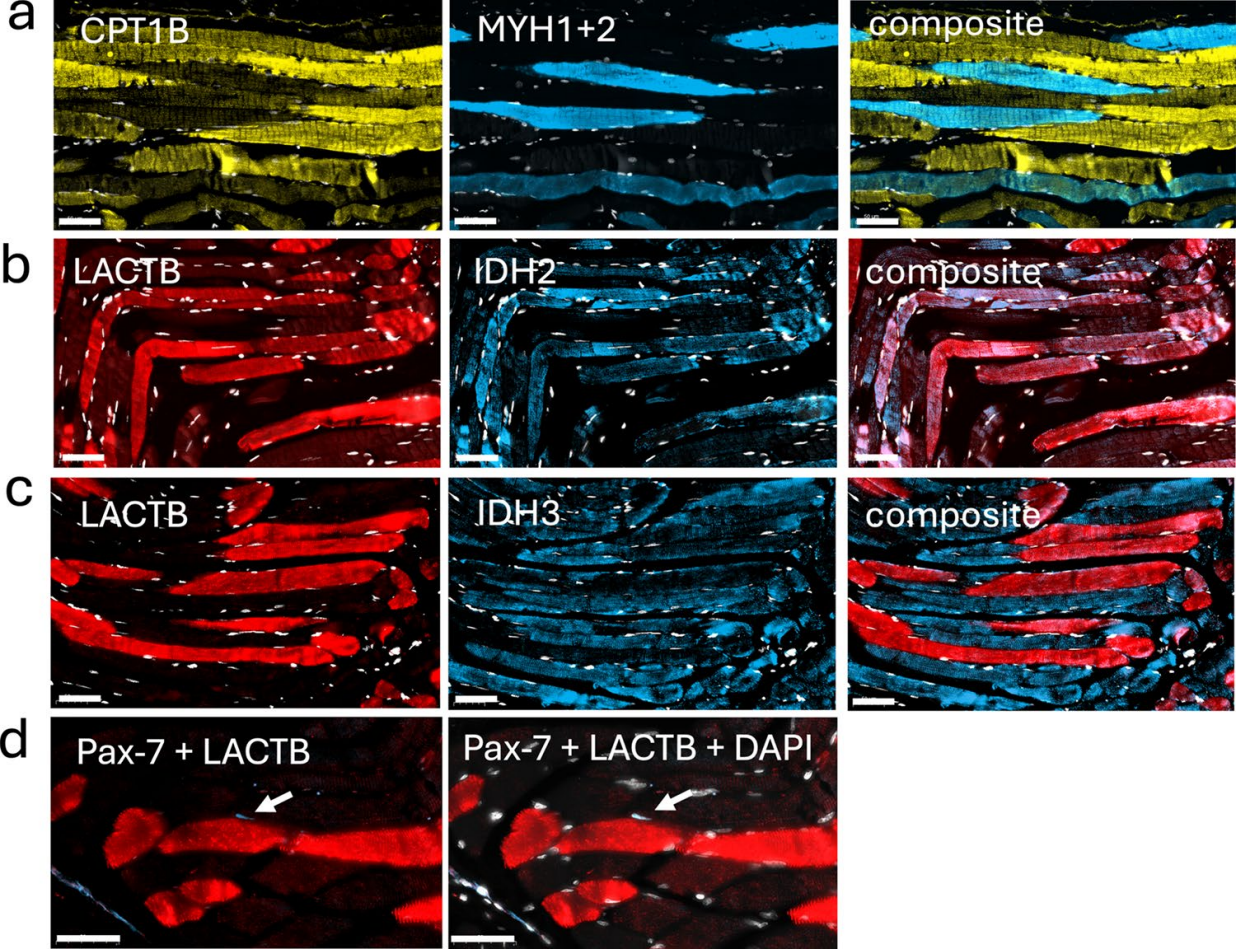
Visualization of LACTB, MYH1+2, metabolic markers, and the paired box protein transcription factor (Pax-7) in adult human muscle. Row a: carnitine palmitoyltransferase 1B (CPT1B) (yellow) and MYH1+2 (blue). Row b: LACTB (red) and isocitrate dehydrogenase 2 (IDH2) (blue). Row c: LACTB (red) and isocitrate dehydrogenase 3 (IDH3) (blue). Row d: LACTB (red) and (Pax-7) (blue). Nuclei stained with DAPI are shown in white. The scale bars are 50 µm

Skeletal muscle is a heterogeneous tissue composed of diverse fiber types that are classified into types I, IIa, IIx, and IIb, based on the expression of myosin heavy chain (MYH) isoforms (Schiaffino and Reggiani 2011). To determine whether LACTB expression correlated with fiber type, we performed double IHC of the quadriceps muscle using the anti-hLACTB antibody and antibodies specific for the different MYH isoforms. The anti-MYH2 antibody that is specific for type IIa fibers and the anti-MYH1 antibody that is specific for type IIx fibers showed a negative correlation with LACTB (Fig. 2, rows a and b). In contrast, the anti-MYH7 antibody that is specific for type I fibers overlapped with LACTB (Fig. 3, row c). These results were corroborated using antibodies against slow fiber troponin T (TnnT1) that is expressed at high levels in type I fibers (Murgia et al. 2015) (Fig. 2, row d). Based on the complement of contractile proteins, we concluded that LACTB-positive fibers were exclusively of type I. Fiber-specific proteome analyses (Murgia et al. 2015) have revealed that the fiber types also differ in terms of their complement of metabolic enzymes, with type I fibers relying more on beta-oxidation than type II fibers. To probe the metabolic nature of the LACTB-positive fibers, we used antibodies against the muscle-specific B isoform of carnitine palmitoyl transferase 1B (CPT1-B), a key component of the mitochondrial fatty acid import system. The results (Fig. 3, row a) revealed that CPT1-B was expressed at a higher level in fibers negative for MYH1 and MYH2, confirming a higher capacity for mitochondrial fatty acid import in the LACTB-positive fibers. Type I and II fibers also differ markedly in their expression of isocitrate dehydrogenase (IDH) isoforms, with IDH2 being expressed predominantly in type I fibers and IDH3 in type II fibers (Murgia et al. 2015; Lang et al. 2018). Double IHC confirmed higher content of IDH2 in LACTB-positive fibers, whereas the content of IDH3 was higher in LACTB-negative fibers (Fig. 3, rows b and c). Since LACTB can reduce the activity of phosphatidylserine decarboxylase (PISD) in some model systems (Keckesova et al. 2017), we investigated the expression of PISD by IHC, but we found no detectable difference between LACTB-positive and LACTB-negative fibers (Fig. S3).

Next, we asked whether LACTB expression is restricted to mature muscle fibers or whether LACTB also occurs in the myogenic stem cells, i.e., in the satellite cells present in adult muscles. To this end, we identified the satellite cells using an antibody against the transcription factor Pax-7, a marker for myogenic stem cells (Fig. 3, row d). This antibody revealed individual Pax-7-positive satellite cells scattered among the mature fibers, which were negative for Pax-7. Double ICH revealed that LACTB was not expressed at a detectable level in satellite cells, indicating that LACTB expression is initiated during the differentiation of myogenic stem cells into mature myofibers.

Having used structural, metabolic, and transcriptional markers as criteria, we conclude that LACTB is expressed predominantly, if not exclusively, in type I oxidative muscle fibers in humans.

### Rat adult skeletal muscle

The muscle fiber types and their properties are not necessarily identical in rodents and humans, exemplified by the almost complete lack of type IIb fibers in humans (Schiaffino and Reggiani 2011). We therefore investigated the skeletal muscle of male adult rats for LACTB expression. Double IHC was performed using the anti-rLACTB antibody and antibodies against MYH1, MYH2, TnnT1, and CPT1B, respectively. The results (Fig. S4) revealed that fibers positive for MYH1 and MYH2 were negative for LACTB, whereas the fibers positive for TnnT1 and CPT1B were also positive for LACTB. In line with the results for human muscle, we conclude that LACTB is expressed predominantly in type I muscle fibers in the rat.

### Human fetal muscle

**Fig. 4:**
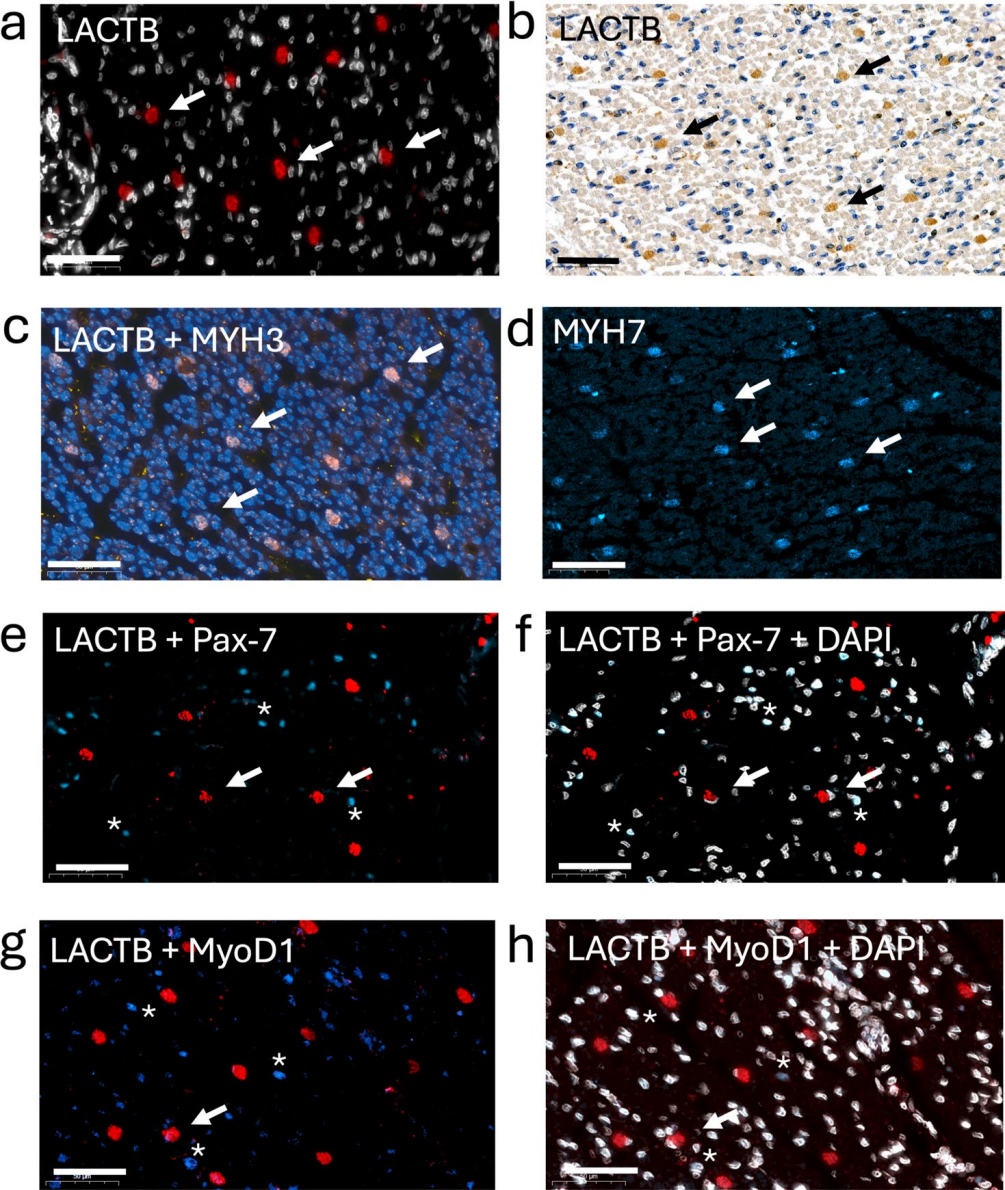
Visualization of LACTB and differentiation markers in human fetal muscle at gestational week 20. Panel **a** LACTB visualized with fluorescence. Nuclei stained with DAPI are shown in white. Panel **b** LACTB visualized with DAB. Panel **c** LACTB (red) and MYH3 (blue). Panel **d** MYH7 (blue). Panels **e** and **f** LACTB (red), Pax-7 (blue), and DAPI (white). Panels **g** and **h** LACTB (red), myoblast determination protein 1 (MyoD1) (blue) and DAPI (white). Scale bars = 50 µm. Examples of LACTB-positive cells are indicated with arrows in each image. Examples of Pax-7 and MyoD1-positive cells are indicated with asterisks in panels **e**–**h**. Note the crescent-shaped Pax-7 and MyoD1-negative nuclei in LACTB-positive cells marked with arrows in panels **f** and **h**

The MYH complement characteristic for the respective fiber types is established during the early postnatal period (Schiaffino and Reggiani 2011). To determine whether LACTB expression commences earlier in the myogenesis, we examined human fetal muscle at gestational week 20. IHC using the anti-hLACTB antibody revealed individual evenly scattered cells, about 15 µm in diameter, surrounded by a population of smaller LACTB-negative cells (Fig. 4, panels a and b). To characterize the LACTB-positive cells, we used antibodies against fetal myosin (MYH3), perinatal myosin (MYH8), MYH7, MYH1, and MYH2, respectively. Both the larger LACTB-positive and the smaller LACTB-negative cells expressed MYH3 (Fig. 4, panel c), while MYH7 was expressed exclusively in the LACTB-positive cells (Fig. 4, panel d). In contrast, MYH8, MYH1, and MYH2 were not expressed at detectable level (results not shown). Testing for the myogenic differentiation markers Pax-7 and MyoD1 revealed that LACTB-positive cells were negative for both markers, while a fraction of the LACTB-negative cells stained positive for Pax-7 (Fig. 4, panels e and f) and MyoD1 (Fig. 4, panels g and h). These findings define the LACTB-positive cells as type I myofibers, which are formed from the primary generation of myotubes, while the surrounding cells are still undifferentiated and will later develop into the secondary generation of myotubes (Romero et al. 2013). Using metabolic markers, we discovered that CPT1B and IDH2 were more abundant in LACTB-positive cells (Fig. S5) than in LACTB-negative cells, while no IDH3 expression was detected in the fetal muscle (not shown). These results show that LACTB is expressed concomitantly with the formation of the primary generation of myotubes which have acquired some metabolic features of adult type I fibers.

### Myoblast differentiation

To substantiate these findings, we investigated LACTB expression during differentiation of mouse C2C12 and rat L6 myoblasts. Confluent C2C12 myoblasts grown on hydrogel were induced to differentiate into multinucleated myotubes by serum starvation (Fig. S6, panel A). Analysis by immunoblotting revealed that LACTB and MYH1 were undetectable in C2C12 myoblasts, while differentiation elicited expression of both LACTB and fast myosins after 3 days (Fig. S6, panel B), in agreement with previous studies (Keckesova et al. 2017). Similarly, L6 myoblasts were allowed to differentiate to myotubes by serum starvation (Fig. S6, panel C). While detectable at low levels already in L6 myoblasts, LACTB expression increased continuously during the differentiation process (Fig. S6, panel D). These results support the conclusions drawn from the IHC studies of fetal muscle showing that LACTB expression begins at an early stage in myotube differentiation. We infer that LACTB is a mitochondrial marker of myogenic differentiation.

## Discussion

The present study shows that LACTB is expressed in a binary manner consistent with the canonical MYH-based classification into type I and II fibers. Fiber-specific quantitative mass spectrometry analyses have revealed a more than tenfold enrichment for the MYH isoforms in the respective fiber types, while for metabolic proteins, less than fivefold differences are generally observed between the fiber types (Murgia et al. 2015; Murgia et al. 2021; Lang et al. 2018; Moreno-Justicia et al. 2025). Based on the difference in staining intensities obtained for the MYH isoforms, we infer that the difference in LACTB expression between type I and type II fibers was at least tenfold at the protein level. Since LACTB is expressed at a high level also in several non-contractile tissues, e.g., liver and adrenal gland, we conclude that the function of LACTB pertains to some specific metabolic process required at substantially higher levels in type I fibers than in type II fibers. Several mitochondrial processes, including fatty acid beta-oxidation, ketone body utilization, and branched-chain amino acid catabolism, are enriched in type I fibers (Murgia et al. 2015; Moreno-Justicia et al. 2025). Our results using metabolic markers confirmed these findings, pointing to a role for LACTB in the setting of mitochondrial fatty acid and lipid turnover, in line with results from other studies (Schadt et al. 2005; Chen et al. 2008; Yang et al. 2009; Keckesova et al. 2017).

Mitochondria exhibit a high degree of functional specialization not only between different cell types but also at the subcellular level, determined by factors including regionalization, metabolic status, and cell cycle phase (Benador et al. 2018; Döhla et al. 2022). Due to the arrangement of the contractile elements, myofibrillar mitochondria are highly restricted in their movement, although their spatial organization differs between the fiber types. While mitochondria in both fiber types are primarily arranged in pairs running parallel with the I-bands, the mitochondria in type I fibers are also interconnected by longitudinally oriented segments extending over several sarcomeres (Mishra et al. 2015; Glancy et al. 2015). In consequence, mitochondria in oxidative fibers form a functionally connected reticulum where processes utilizing proton-motive force are enriched in the I-band-associated mitochondria, whereas generation of membrane potential takes place predominantly in mitochondria located in the longitudinal and peripheral parts (Glancy et al. 2015). Therefore, the predominant location of LACTB in I-band-associated mitochondria suggests a function related to ATP production rather than to processes generating proton-motive force. The implications of this finding merit further investigation in future studies.

Skeletal muscle is quantitatively the most important target organ of insulin, and accounts for 75% of the postprandial glucose uptake. Dysregulation of this function leads to insulin resistance and type 2 diabetes through multifactorial mechanisms. Diabetes is associated with myopathy, abnormal lipid accumulation (Levin et al. 2001), impaired mitochondrial respiration and enzyme dysregulation (Morgensen et al. 2007; Ritov et al. 2010), and reduced mitochondrial quantity, including an altered ultrastructure (Kelley et al. 2002). Type I fibers have both a higher capacity for glucose handling and a higher glucose uptake in response to stimulation by insulin than type II fibers (Stuart et al. 2013; Cartee et al. 2016). Notably, type I and type II fibers are affected differently during the progression towards type 2 diabetes, resulting in a fiber type shift towards a lower proportion of type I fibers (Stuart et al. 2013; Pataky et al. 2017). Our findings, indicating that LACTB is distinctive for the metabolic profile of type I fibers, raise the question as to whether a decreased function of LACTB may contribute to a selective decline in the type I fiber population and hence to the fiber type shift seen in diabetes. This notion was substantiated in a recent study on LACTB knockout mice, which showed impaired glucose tolerance and elevated blood lipid level (Li 2024), revealing a potential link between LACTB and the diabetic process.

LACTB has evolved from bacterial PBP-βLs (Peitsaro et al. 2008) which participate in the synthesis and maintenance of peptidoglycan, a cellular constituent lacking in eukaryotic organisms. Therefore, LACTB must have been converted for a different biochemical purpose in early eukaryotes. The present findings indicate that LACTB plays a role in the differentiation of energy metabolism during muscle fiber development towards a more oxidative phenotype. LACTB is generally downregulated in tumors, which may reflect a shift in the metabolism from an oxidative fatty acid-utilizing metabolism to a glucose-dependent metabolism, in line with the classical Warburg effect. Further studies on the biochemical and physiological roles of LACTB in various tissues may generate novel insight into the function of mitochondria in normal and pathological processes.

## Supplementary Information

Below is the link to the electronic supplementary material.

Supplementary file1 (DOCX 26 KB)

Supplementary file2 (DOCX 19 KB)

Supplementary file3 (MP4 12580 KB) Video S1 3D rendering of the distribution of LACTB within the mitochondrial network, pseudo-colored in green. The red channel shows the distribution of COX-IV, with the intensity lowered for clarity

Supplementary file4 (TIFF 65900 KB)

Supplementary file5 (TIFF 65900 KB)

Supplementary file6 (TIFF 41774 KB)

Supplementary file7 (TIFF 65900 KB)

Supplementary file8 (TIFF 41774 KB)

Supplementary file9 (TIFF 65900 KB)

## Data Availability

No datasets were generated or analyzed during the current study.

## List of Abbreviations

CPT1-B: Carnitine palmitoyl transferase IB
DAB: 3,3-Diaminobenzidine
DAPI: 4′,6-Diamidino-2-phenylindole
DMSO: Dimethyl sulfoxide
hLACTB: Human LACTB
IDH: Isocitrate dehydrogenase
IHC: Immunohistochemistry
MYH: Myosin heavy chain
PBS: Phosphate-buffered saline
PISD: Phosphatidyl serine decarboxylase
rLACTB: Rat LACTB
RT: Room temperature
TBST: Tris-buffered saline containing 0.05% Tween 20
TnnT1: Slow fiber troponin T
VDAC1: Voltage-dependent anion channel 1
