# Supplementary material for: Fiber type-specific expression of LACTB leverages a function in oxidative metabolism
Source: Histochem Cell Biol. 2026 Apr 18;164(1):24. doi: 10.1007/s00418-026-02476-8 (PMC13091871; doi:10.1007/s00418-026-02476-8)
Supplement: Supplementary file 1 — Supplementary file1 (DOCX 26 KB) [file 418_2026_2476_MOESM1_ESM.docx]

Fiber type-specific expression of LACTB leverages a function in oxidative metabolism

Histochemistry and Cell Biology

Alanen K-A, Soliymani R, Sarparanta J, Kuure S, Sainio K, Polianskyte Z, Asghar MY, Zangene E, Cascone A, Lalowski M, Hackman P, Lundin J, Lindholm D, Eriksson O

Corresponding author:

Ove Eriksson, University of Helsinki, Finland

Email: ove.eriksson@helsinki.fi

**Supplementary table 1.** List of commercially available primary and secondary antibodies used including literature references.

| Target | Manufacturer | Catalogue # | Host | RRID | References |
| --- | --- | --- | --- | --- | --- |
| COX-IV | Proteintech | 66110-1-Ig | Mouse | AB_2881509 | (1) |
| CPT1B | Proteintech | 22170-1-AP | Rabbit | AB_2713959 | (2) |
| GAPDH | Cell Signaling | 97166S | Mouse | AB_2756824 | (3) |
| IDH-2 | Abcam | ab55271 | Mouse | AB_943793 | (4) |
| IDH-3 | Proteintech | 68199-1-Ig | Mouse | AB_2935288 | (5) |
| Ki-67 | Abcam | ab279653 | Mouse | AB_2934265 | (6) |
| Laminin beta 1 | Merck | MAB1921b | Mouse | AB_571039 | (7) |
| MYH1 | Proteintech | 67299-1-Ig | Mouse | AB_2882563 | (8) |
| MYH2 | Proteintech | 66212-1-Ig | Mouse | AB_2881603 | (9) |
| MYH3 | Invitrogen | PA5-72848 | Rabbit | AB_2718702 | (10) |
| MYH7 | Invitrogen | MA1-83347 | Mouse | AB_936651 | (11) |
| MYH8 | Invitrogen | PA5-72846 | Rabbit | AB_2718700 | (12) |
| MyoD1 | Abcam | ab16148 | Mouse | AB_2148758 | (13) |
| Pax-7 | Abcam | ab218472 | Mouse | n/a | (14) |
| PISD | Abcam | ab236405 | Mouse | n/a | (15) |
| TNNT1 | Proteintech | 68631-1-Ig | Mouse | AB_3085322 | (16) |
| VDAC1 | Merck | MABN504 | Mouse | AB_2890972 | (17) |
| Anti-mouse Ig HRP | Invitrogen | G-21040 | Goat | AB_2536527 | (18) |
| Anti-rabbit Ig HRP | ThermoFisher | A16035 | Donkey | AB_2534709 | (19) |
| Anti-rabbit Ig Alexa-647 | Life | A21245 | Goat | AB_2535813 | (20) |
| Anti-mouse Ig Alexa-750 | Life | A21037 | Goat | AB_2535708 | (21) |

(1) Dong, J., Chen, L., Ye, F. *et al.* Mic19 depletion impairs endoplasmic reticulum-mitochondrial contacts and mitochondrial lipid metabolism and triggers liver disease. *Nat Commun* **15**, 168 (2024). <https://doi.org/10.1038/s41467-023-44057-6>

(2) Simcox, J., Geoghegan, G., Maschek, J. A., et al. Global analysis of plasma lipids identifies liver-derived acylcarnitines as a fuel source fro brown fat thermogenesis. *Cell Metabol* **26(3),** 509-522 (2017).

<https://doi.org/10.1016/j.cmet.2017.08.006>

(3) Pandovski S, Yang T, Zhou H, Rosahl TW, Carballo-Jane E, Talukdar S, Coyne ES. A mitochondrial amidoxime-reducing component 1 (mARC1) A168T amino acid substitution does not confer protection from MASH and fibrosis in multiple mouse models of chronic liver disease. *Biochem J.* **483(2)**:BCJ20253411 (2026).

<https://doi.org/10.1042/BCJ20253411>

(4) Li, Jj., Yu, T., Zeng, P. *et al.* Wild-type IDH2 is a therapeutic target for triple-negative breast cancer. *Nat Commun* **15**, 3445 (2024).

<https://doi.org/10.1038/s41467-024-47536-6>

(5) Zhang S, Sha Y, Xie Y, Hong J. Kaempferol Alleviates Dry Eye Disease Via Modulation of the IDH3B/CCAR2/IKBKB Axis. *Invest Ophthalmol Vis Sci.* **66(12)**, 50 (2025)

<https://doi.org/10.1167/iovs.66.12.50>

(6) Kostin, A.; Lyundup, A.; Alekhnovich, A.; Prikhodko, A.; Patsap, O.; Gronskaia, S.; Belaya, Z.; Lesnyak, O.; Melnichenko, G.; Mokrysheva, N.; et al. Mast Cell Association with the Microenvironment of a Phosphaturic Mesenchymal Tumour Secreting Fibroblast Growth Factor 23. *Med Sci*, **13**, 195 (2025).

<https://doi.org/10.3390/medsci13030195>

(7) Fan J, Liu Y, Wang C, Feng Z, Pan J, Peng Y, Peng J, Bao Y, Nie J, Qiu B, Qi S. Reinvestigating Tumor-Ventricle Relationship of Craniopharyngiomas With Predominantly Ventricular Involvement: An Endoscopic Endonasal Series Based on Histopathological Assessment. *Front Oncol.* **11**:740410 (2021)

<https://doi.org/10.3389/fonc.2021.740410>

(8) Luo, X.; Zhang, H.; Cao, X.; Yang, D.; Yan, Y.; Lu, J.; Wang, X.; Wang, H. Endurance Exercise-Induced Fgf21 Promotes Skeletal Muscle Fiber Conversion through TGF-β1 and p38 MAPK Signaling Pathway.  *Int J Mol Sci,* **24**, 11401 (2023)

<https://doi.org/10.3390/ijms241411401>

(9) Zhang, W., You, B., Qi, D. *et al.* Trimetazidine and exercise provide comparable improvements to high fat diet-induced muscle dysfunction through enhancement of mitochondrial quality control. *Sci Rep* **11**, 19116 (2021).

<https://doi.org/10.1038/s41598-021-98771-6>

(10) Blain, R., Coulu, G., Shotar, E. *et al.* A tridimensional atlas of the developing human head. *Cell*

**186(26)**, 5910-5924.e17 (2023).

<https://doi.org/10.1016/j.cell.2023.11.013>

(11) Xue H, Shi H, Zhang F, Li H, Li C, Han Q. RIP3 Contributes to Cardiac Hypertrophy by Influencing MLKL-Mediated Calcium Influx. *Oxid Med Cell Longev.* **2022**:5490553 (2022).

<https://doi.org/10.1155/2022/5490553>

(12) Hennig K, Hardman D, Barata DM, Martins II, Bernabeu MO, Gomes ER, Roman W. Generating fast-twitch myotubes in vitro with an optogenetic-based, quantitative contractility assay. *Life Sci Alliance*. **6(10)**:e202302227 (2023).

<https://doi.org/10.26508/lsa.202302227>

(13) Dandan Tan, Yidan Liu, Huaxia Luo, Qiang Shen, Xingbo Long, Luzheng Xu, Jieyu Liu, Nanbert A Zhong, Hong Zhang, Hui Xiong. A novel mouse model for LAMA2-related muscular dystrophy with analysis of molecular pathogenesis and clinical phenotype. *eLife*, **13**:RP94288 (2025)

<https://doi.org/10.7554/eLife.94288.4>

(14) Wirtz J, Chaney R, Cefis M, Méloux A, Wang Y, Lemaire S, Quirié A, Delezie J, Gouspillou G, Prigent-Tessier A, Garnier P. Electrical Stimulation-Induced Muscle Damage Alters Hippocampal BDNF Signaling. Eur J Neurosci. **62(6)**, e70235 (2025).

<https://doi.org/10.1111/ejn.70235>

(15) Luo Y, Zhang Y, Pang S, Min J, Wang T, Wu D, Lin C, Xiao Z, Xiang Q, Li Q, Ma L. PCBP1 protects bladder cancer cells from mitochondria injury and ferroptosis by inducing LACTB mRNA degradation. *Mol Carcinog.* **62(7)**:907-919 (2023).

<https://doi.org/10.1002/mc.23533>

(16) Laarne M, Oghabian A, Laitila J, Isohanni P, Tynninen O, Zhao F, Rostedt F, Sarparanta J, Sagath L, Lawlor MW, Wallgren-Pettersson C, Lehtokari VL, Pelin K. A homozygous single-nucleotide variant in *TNNT1* causes abnormal troponin T isoform expression in a patient with severe nemaline myopathy: A case report. *J Neuromuscul Dis.* **12(5)**:689-698 (2025).

<https://doi.org/10.1177/22143602251339569>

(17) Madungwe NB, Feng Y, Imam Aliagan A, Tombo N, Kaya F, Bopassa JC. Inner mitochondrial membrane protein MPV17 mutant mice display increased myocardial injury after ischemia/reperfusion. *Am J Transl Res.* **12(7)**:3412-3428 (2020)

PMID: 32774709

(18) Son, S., Xu, C., Fu, H. *et al.* Neutrophils preserve energy storage in sympathetically activated adipocytes. *Nature* **650**, 718–726 (2026).

<https://doi.org/10.1038/s41586-025-09839-6>

(19) Bell L, Clerkin S, Rizalar S, Rizkallah A, Stokar-Regenscheit N, Spijkers XM, Wevers NR, Simonneau C, Augustin A, Höllbacher B, D'Abate L, Ficek-Pascual J, Schneider K, Von Tell D, Maurissen T, Zanini C, Zundel C, Golling S, Becker C, Odermatt A, Foo LC, Pigoni M, Villaseñor R. ApoE4 disrupts intracellular trafficking and iron homeostasis in a reproducible iPSC-based model of human brain endothelial cells. *Stem Cell Reports.* **20(9)**:102607 (2025)

<https://doi.org/10.1016/j.stemcr.2025.102607>

(20) Abe T, Murao A, Yamaga S, Wang P, Aziz M. B-1a cells mitigate radiation injury by protecting intestinal barrier integrity. *Front Immunol.* **17**:1761007 (2026).

<https://doi.org/10.3389/fimmu.2026.1761007>

(21) Felix-Lopez, A., Lopez-Orozco, J., Elish, M. *et al.* FGFR signaling and neddylation facilitate SARS-CoV-2 infection by modulating interferon induction and vital entru, respectively. *iScience*, **29(2)**, 114566 (2926)

<https://doi.org/10.1016/j.isci.2025.114566>
