# Supplementary material for: Fiber type-specific expression of LACTB leverages a function in oxidative metabolism
Source: Histochem Cell Biol. 2026 Apr 18;164(1):24. doi: 10.1007/s00418-026-02476-8 (PMC13091871; doi:10.1007/s00418-026-02476-8)
Supplement: Supplementary file 2 — Supplementary file2 (DOCX 19 KB) [file 418_2026_2476_MOESM2_ESM.docx]

Fiber type-specific expression of LACTB leverages a function in oxidative metabolism

Histochemistry and Cell Biology

Alanen K-A, Soliymani R, Sarparanta J, Kuure S, Sainio K, Polianskyte Z, Asghar MY, Zangene E, Cascone A, Lalowski M, Hackman P, Lundin J, Lindholm D, Eriksson O

Corresponding author:

Ove Eriksson, University of Helsinki, Finland

Email: ove.eriksson@helsinki.fi

**Supplementary table 2.** Relative expression level of *LACTB* mRNA in various human adult and fetal tissues.

| **Position** | **Tissue** | **Dot intensity, %** |
| --- | --- | --- |
| 1A | whole brain | 48 |
| 1B | cerebral cortex | 44 |
| 1C | frontal lobe | 45 |
| 1D | pariental lobe | 46 |
| 1E | occipital lobe | 42 |
| 1F | temporal lobe | 54 |
| 1G | postcentral gyrus of cerebral cortex | 31 |
| 1H | pons | 23 |
| 2A | cerebellum, left | 57 |
| 2B | cerebellubm, right | 58 |
| 2C | corpus callosum | 45 |
| 2D | amygdala | 38 |
| 2E | caudate nucleus | 72 |
| 2F | hippocampus | 39 |
| 2G | medulla oblongata | 32 |
| 2H | putamen | 26 |
| 3A | subbstantia nigra | 42 |
| 3B | accumbens nucleus | 49 |
| 3C | thalamus | 24 |
| 3D | pituitary gland | 43 |
| 3E | spinal cord | 37 |
| 4A | heart | 100 |
| 4B | aorta | 31 |
| 4C | atrium, left | 53 |
| 4D | atrium, right | 46 |
| 4E | ventricle, left | 75 |
| 4F | ventricle, right | 61 |
| 4G | interventricular septum | 69 |
| 4H | apex of the heart | 43 |
| 5A | esophagus | 37 |
| 5B | stomach | 52 |
| 5C | duodenum | 41 |
| 5D | jejunum | 39 |
| 5E | ileum | 18 |
| 5F | ilocecum | 15 |
| 5H | appendix | 24 |
| 5A | colon, ascending | 37 |
| 6A | colon, transverse | 48 |
| 6B | colon, desending | 34 |
| 6C | rectum | 38 |
| 7A | kidney | 61 |
| 7B | skeletal muscle | 99 |
| 7C | spleen | 43 |
| 7D | thymus | 20 |
| 7E | peripheral blood leukocyte | 39 |
| 7F | lymph node | 52 |
| 7H | trachea | 11 |
| 8A | lung | 48 |
| 8B | placenta | 34 |
| 8C | bladder | 19 |
| 8D | uterus | 15 |
| 8E | prostate | 25 |
| 8F | testis | 47 |
| 8G | ovary | 3 |
| 9A | liver | 79 |
| 9B | pancreas | 41 |
| 9C | adrenal gland | 40 |
| 9D | thyroid gland | 18 |
| 9E | salivary gland | 21 |
| 9F | mammary gland | 0 |
| 10A | leukemia, HL-60 | 23 |
| 10B | HeLa S3 | 42 |
| 10C | leukemia, K-562 | 19 |
| 10D | leukemia, MOLT-4 | 5 |
| 10E | Burkitt's lymphoma, Raji | 3 |
| 10F | Burkitt's lymphoma, Daudi | 0 |
| 10G | colorectal adenocarcinoma, SW480 | 0 |
| 10H | lung carcinoma, A549 | 0 |
| 11A | fetal brain | 32 |
| 11B | fetal heart | 52 |
| 11C | fetal kidney | 30 |
| 11D | fetal liver | 28 |
| 11E | fetal spleen | 19 |
| 11F | fetal thymus | 5 |
| 11G | fetal lung | 34 |
| 12A | yeast total RNA | 0 |
| 12B | yeast tRNA | 0 |
| 12C | *E. coli* rRNA | 0 |
| 12D | *E. coli* DNA | 0 |
| 12E | Poly r(A) | 0 |
| 12F | human c_0_t-1 DNA | 0 |
| 12G | human DNA 100 ng | 0 |
| 12H | human DNA 500 ng | 0 |
