# Supplementary figures and images for: Fiber type-specific expression of LACTB leverages a function in oxidative metabolism
Source: Histochem Cell Biol. 2026 Apr 18;164(1):24. doi: 10.1007/s00418-026-02476-8 (PMC13091871; doi:10.1007/s00418-026-02476-8)

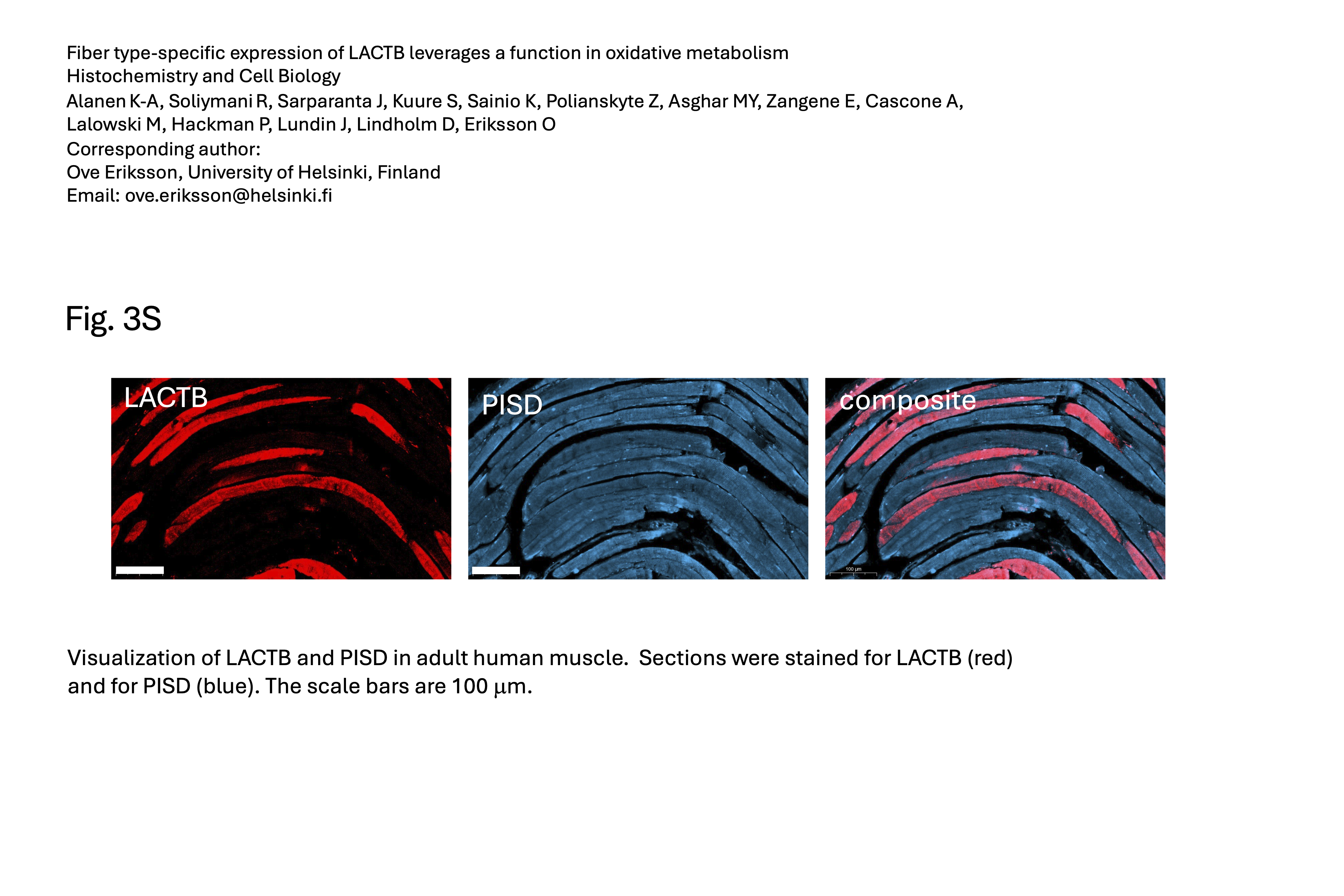

Supplement: Supplementary file 6 — Supplementary file6 (TIFF 41774 KB) [file 418_2026_2476_MOESM6_ESM.tiff]

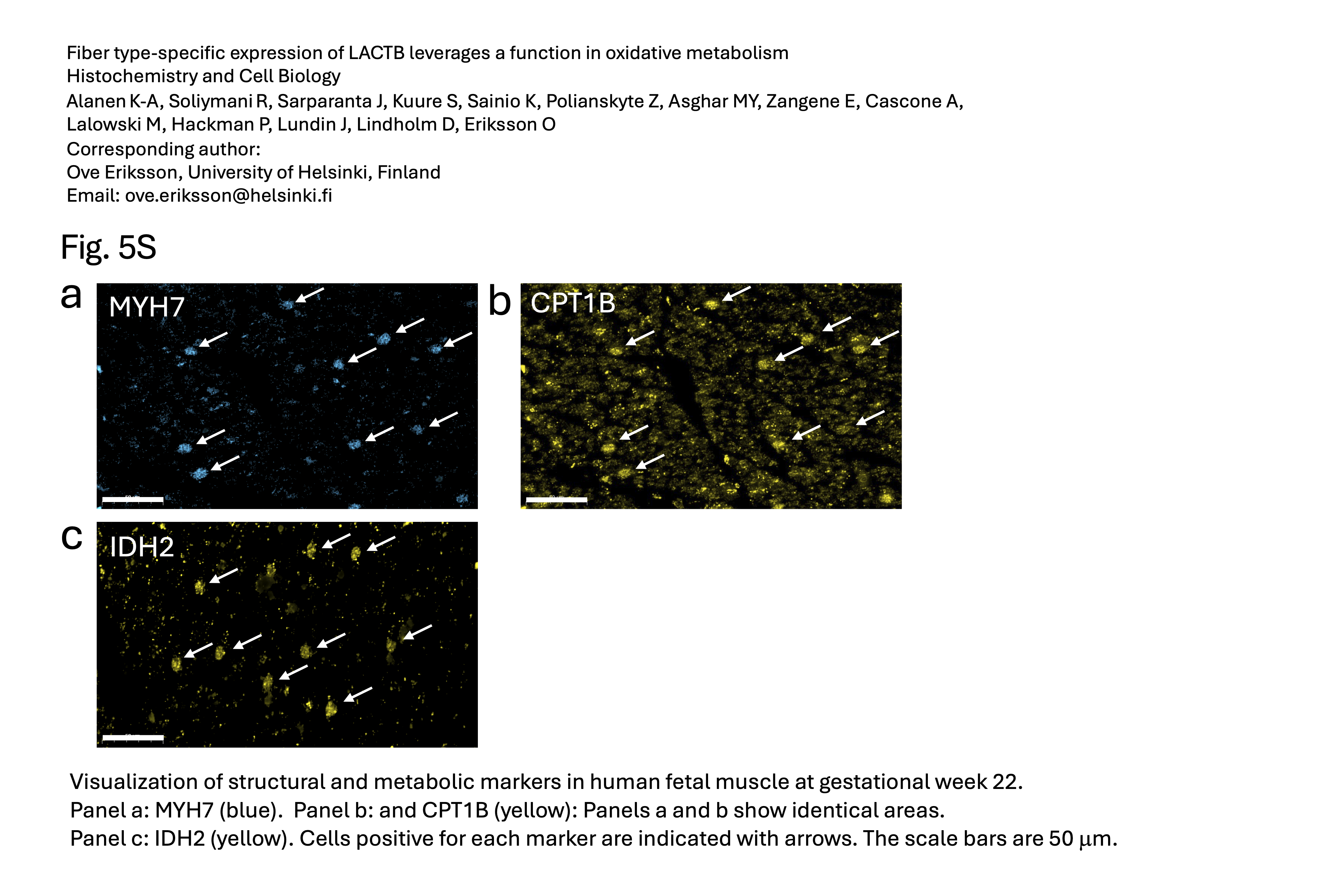

Supplement: Supplementary file 8 — Supplementary file8 (TIFF 41774 KB) [file 418_2026_2476_MOESM8_ESM.tiff]
